# Clinical evaluation following the use of mineralized collagen graft for bone defects in revision total hip arthroplasty

**DOI:** 10.1093/rb/rbv022

**Published:** 2015-11-11

**Authors:** Cheng Huang, Liwu Qin, Wei Yan, Xisheng Weng, Xiangjie Huang

**Affiliations:** ^1^Department of Orthopaedics, Peking Union Medical College Hospital, Chinese Academy of Medical Sciences and Peking Union Medical College, Beijing 100730, China and; ^2^Department of Orthopaedics, Wendeng Orthopaedic Hospital of Shandong Province Affiliated to Shandong University of Traditional Chinese Medicine, Weihai 264400, China

**Keywords:** total hip arthroplasty, revision, bone defect, mineralized collagen, bone substitutes

## Abstract

Revision total hip arthroplasty (THA) with massive bone loss has been a real challenge for orthopaedic surgeons. Here we describe an approach using mineralized collagen (MC) graft to reconstruct acetabulum and femur with massive bone defects. We identified 89 patients suffering acetabular or femoral bone defects after primary THA, who required revision THA for this study. During the surgery, MC was applied to reconstruct both the acetabular and femoral defects. Harris hip score was used to evaluate hip function while radiographs were taken to estimate bone formation in the defect regions. The average follow-up period was 33.6 ± 2.4 months. None of the components needed re-revised. Mean Harris hip scores were 42.5 ± 3.5 before operation, 75.2 ± 4.0 at 10th month and 95.0 ± 3.6 at the final follow-up. There were no instances of deep infection, severe venous thrombosis or nerve palsy. The present study demonstrated that MC graft can serve as a promising option for revision THA with massive bone deficiency. Meanwhile, extended follow-up is needed to further prove its long-term performance.

## Introduction

Total hip arthroplasty (THA) has become one of the most common and effective methods to treat severe hip diseases. In recent years, an increasing number of revision THA has been observed along with the growing number of primary THA. Massive bone defects can lead to aseptic loosening and hip arthroplasty osteolysis, composing the two main reasons for revision THA (69 ± 3.5%) [1, 2]. Revision to a failed THA is a truly challenge to orthopaedic surgeons and normally requires hospitals with high level in the presence of massive bone defects [3]. Reconstruction of the bone defects can be directly related to the post-revision stability and service life of prosthesis [4]. Currently, the main approaches to repair bone defects are autologous and xenogenous bone grafts. Although autologous bone graft is an ideal substitute, limited sources and extra pain from getting autologous bone graft restricted its development. While allograft bone can avoid such issue, it can lead to potential risk of biological immune rejection and is not favorable for bone growth as a result of biological incompatibility [3, 5].

**Figure 1.**
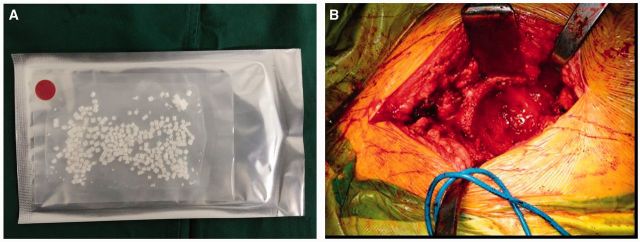
Commercial mineralized collagen were demonstrated in (A). Slightly grinded acetabulum was shown in (B), where mineralized collagen grafts were implanted to repair bone defects

Tissue engineering scaffolds play a vital role in regenerative medicine [6, 7]. It not only provides a temporary 3-dimensional support during tissue repair, but also regulates the cell behavior, such as cell adhesion, proliferation and differentiation. As a kind of bone-resembling material (Fig. 1A), MC graft demonstrated bioactive and biodegradable properties both in vitro and in vivo [8–11]. Numerous studies have confirmed that porous MC scaffold can provide a mimic in vivo microenvironment, in which osteogenic cells eventually developed into tridimensional polygonal shape. In addition, new bone matrix was synthesized at the interface of bone fragments and within the composite [12]. Here we retrospected our patients suffering revision THA with MC composite grafts after midterm follow-up and evaluated the performance of MC through hip function and major complications.

## Patients and methods

### Patients

This study was approved by the ethics committee of Wendeng Orthopaedic Hospital of Shandong Province. Written informed consents were obtained from all participants.

Between February 2010 and June 2013, we used MC graft (Beijing Allgens Medical S&T Company) in revision hip arthroplasty for 89 patients caused by aseptic loosening with acetabular or femoral bone defects, including 30 cases with acetabular loosening, 20 cases with femoral loosening and 39 cases with both of them.

The average age of primary hip arthroplasty was 58 (range, 54–75) years and the average time from primary THA to revision THA was 6.9 (range, 3–11) years. All the patients were followed up through outpatient after the surgery. Depending on the roentgenographic and intraoperative findings on the acetabulum and femur, the deficiency was classified according to the system of Paprosky classification (Table 1).

**Table 1.** Case number according to Paprosky classification

	Paprosky classification							
	I	II	IIA	IIB	IIC	IIIA	IIIB	IV
*Cases with*								
Acetabular defects	3	/	10	20	19	10	7	/
Femoral defects	2	10	/	/	/	24	20	3

### Preoperative preparation

Routine examinations were performed to figure out the location, size and scope of bone defects, including anteroposterior radiographs of pelvis, anteroposterior and lateral radiographs of the femur, hip CT scan and three-dimensional reconstruction graphs. Blood cell analysis, C-reactive protein (CRP), erythrocyte sedimentation rate (ESR) and calcitonin were tested to rule out infections.

### Surgical procedures

All revision procedures were performed by one senior surgeon. The posterolateral approach was chosen in all patients. Granulation tissues, cement, particles and interface membranes were thoroughly removed from the acetabular defect region after the exposure and removal of the failed implant. The severity of the bone defect and the periacetabular bone quality were reevaluated under direct observation. After slightly grinding the acetabulum, MC graft was implanted to repair bone defects (Fig. 1B). If the acetabulum ring was damaged, acetabular cup could not obtain initial stability. In cases of this situation, titanium mesh or cage was applied to rebuild stability of acetabulum ring, then followed by MC graft. The acetabulum cup was put finally. In the meantime, femoral handle prosthesis was pulled out by special equipment to make a thorough clean of the bone marrow cavity. Grooves could be made if the bone cement was difficult to take out. We used proximal or distal fixed prosthesis type according to the defect sites. Similarly, MC grafts were implanted to repair bone defects.

### Post-operative care and assessment

Drainage tubes were indwelled for 24 h and standard antibiotics were administered for three days postoperatively. Low molecular weight heparin was subcutaneously injected for 7 days to prevent deep venous thrombosis. Partial weight-bearing with a walker was advised for the first 2 weeks after the surgery, and was followed by progressive weight-bearing with crutches. Free ambulation was allowed after three months. Clinical and radiographic follow-up examinations were performed at 3 months, 6 months, 1 year and then annually after operation. The Harris hip score was used to evaluate the overall hip function.

### Statistical methods

All of the results are presented as the mean ± standard deviation (SD). Statistical analyses were performed using SPSS version 11 (SPSS Inc., Chicago, IL, USA). Student *t*-test was used to compare paired variables. A *P* value < 0.05 was considered significant.

## Results

**Figure 2.**
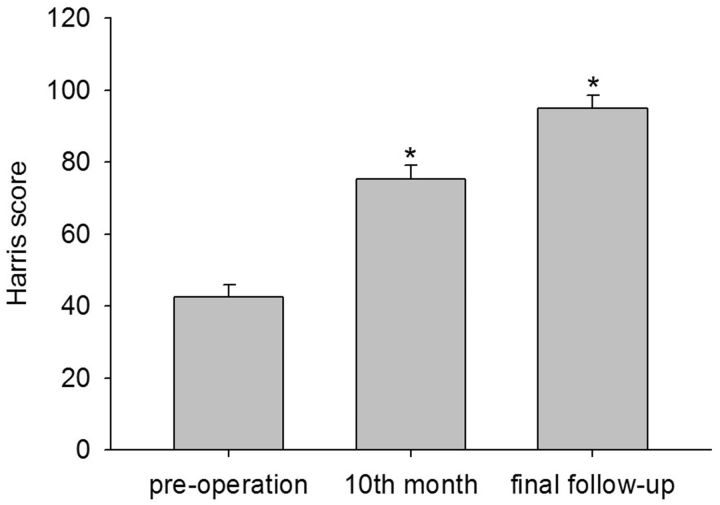
The Harris hip scores were 42.5 ± 3.5 pre-operation, 75.2 ± 4.0 at 10th month and 95.0 ± 3.6 at the final follow-up. The scores of adjacent periods were compared, and the difference were statistically significant (t1 = 9.61, t2 = 7.90, * *P *< 0.01) compared with the scores of adjacent periods

All revision surgeries were successfully completed. No infection, bleeding, prosthetic loosening, implant failure or other major complications occurred during hospital stay. The average follow-up time was 33.6 ± 2.4 (range, 24–53) months. None of the components had been re-revised. There were no instances of deep infection, severe venous thrombosis and nerve palsy throughout the follow-up period. The Harris hip score were 42.5 ± 3.5 pre-operation, 75.2 ± 4.0 at 10th month and 95.0 ± 3.6 at the final follow-up (Fig. 2). The scores of adjacent periods were compared, and the differences were statistically significant (t1 = 9.61, t2 = 7.90, *P *< 0.01).

**Figure 3.**
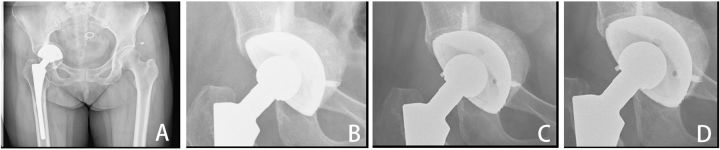
A 58-year-old woman developed prosthetic cup loosening 4 years after THA. Pre-operative radiograph showed massive acetabular bone defect (A). MC grafts were used to reconstruct the acetabulum and radiographs taken at 3rd month (B), 6th month (C) and final follow-up (D) showed the bone grafts were well remodeled.

**Figure 4.**
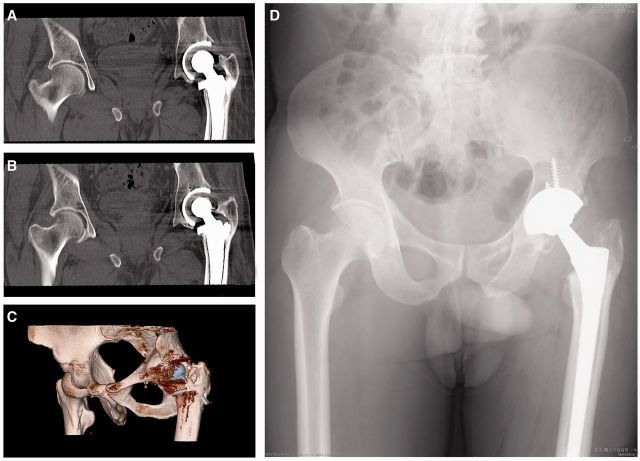
A 65-year-old man developed femoral components loosening 6 years after the operation. Hip CT scan and three-dimensional reconstruction graphs showed bone defects of proximal femur (A–C). MC grafts were used to reconstruct the bone defects and radiographs taken at 3rd month (lost) and 12th month (D) showed that the proximal femur bone grafts were well remodeled

Representative pre-operative and post-operative radiographs were shown in Figs 3 and 4. Bone defects of acetabulum (Fig. 3) and proximal femur (Fig. 4) were reconstructed completely by MC grafts. Flexion activities of all hips were well functioned, and no hip prosthesis loosening was observed (Fig. 3). By the time of the last follow-up, we found that there was no difference between postoperative and last follow-up in terms of acetabulum abduction angle. Hip center of rotation was determined by comparing the operated hips immediately, 3 months, 6 months and 1 year post-revision, no difference was found at horizontal direction or vertical direction between postoperation and last follow-up.

## Discussion

Acetabular and femoral bone defects are commonly existed in failed THA, significantly increase the difficulty of revision surgery [3, 13]. The primary objective of revision surgery is to obtain a stable, durable reconstruction. Secondary objectives include reconstituting the pelvis, stocking femur bone, restoring the hip rotation center to the anatomical location, and minimizing leg-length discrepancies. Complete bone tissue support is necessary to restore the initial stability and ideal long-term effect. Therefore, bone defects reconstruction directly determines the success of THA revision [14]. Unfortunately, the objectives of restoring or preserving pelvic bone stock, placing the acetabular component to the correct anatomic position to optimize joint stability, equalize leg lengths, and achieve stable fixation are not readily achieved in such a situation [3, 15, 16]. Currently, the methods of bone defects reconstruction mainly include impaction of bone particles graft [17] and structural bone grafts [18]. Autogenous bone and allograft bone are the common source of bone graft. Autogenous bone is an ideal bone graft material as a result of its osteoinductive and osteoconductive capabilities [3, 15]. Although autogenous bone could provide osteogenic cells and has neither the immunogenicity nor the risk of disease transmission, its application is significantly restricted by its single source and limited bone mass, especially in the situation when patients suffer massive bone defects. Allogeneic bone graft is the most widely used material currently [15]. Its advantages range from abundant and readily available to obtain to well-behaved initial strength. Nevertheless, osteogenic cell deficiency, biological incompatibility, potential immunogenicity and risk of infectious diseases limit its application [20].

MC graft is composed of hydroxyapatite and type I collagen [21]. The inorganic phase in the composite is carbonate substituted hydroxyapatite with a low crystallinity. The mineral precipitates with a crystal size on a nanometer scale are uniformly distributed on the collagen matrix [12]. The composite, to a large extent, mimics the natural bone environment in both composition and microstructure. A number of in vitro and in vivo studies has proved the bioactive and biodegradable characteristics of this bone-resembling material [22, 23].

As indicated by Ripamonti and Duneas [24], an ideal biomaterial for bone tissue engineering should be nonimmunogenic, biodegradable, highly effective in osteoinduction with relatively low doses of inducing signals, ready for rapid vascularization and mesenchymal cell invasion, carvable, and amenable to contouring for optimal adaptation to the various shapes of bone defects which could provide mechanical support when needed. The MC graft meets nearly the whole requirements as a suitable alternative for bone tissue engineering [12]. In our study, we used MC graft to reconstruct acetabulum and femur with massive bone defects. It provided strong joint stability by restoring the bone defects to fit the acetabular component in the correct anatomic position. Midterm evaluation of hip function showed a full recovery compared to the pre-operation status. Moreover, no major complications such as deep infection, severe venous thrombosis or nerve palsy were observed.

In the same time, our study is also inevitably associated with some limitations. Firstly, this is a retrospective study and lacks control group. Another limitation is the relatively short follow-up time. Although previously published data indicated that long-term (13 years) prosthesis survival and function following revision arthroplasty with a 50/50 mixture of allograft and hydroxyapatite were comparable to allograft alone [25], it is not powerful enough to draw the conclusion among different studies, follow-up evaluation should be continued to enhance the strength of the study. Thirdly, incorporation of the MC graft was only assessed through radiograph, while CT scan of the hip is probably a better solution to define incorporation despite possible metal artifacts.

In conclusion, even though reconstruction of the acetabula and femur with massive defects is still a major surgical challenge in revision THA, our findings manifest that MC graft may be a promising solution for THA revision with massive bone deficiency due to its excellent performance in hip function improvement and few complications. Extended follow-up is necessary to further prove the feasibility of this approach.
